# The Skin in Celiac Disease Patients: The Other Side of the Coin

**DOI:** 10.3390/medicina55090578

**Published:** 2019-09-09

**Authors:** Ludovico Abenavoli, Stefano Dastoli, Luigi Bennardo, Luigi Boccuto, Maria Passante, Martina Silvestri, Ilaria Proietti, Concetta Potenza, Francesco Luzza, Steven Paul Nisticò

**Affiliations:** 1 Digestive Physiopathology Unit, Department of Health Sciences, Magna Graecia University of Catanzaro, 88100 Catanzaro, Italy; 2 Dermatology Unit, Department of Health Sciences, Magna Graecia University of Catanzaro, 88100 Catanzaro, Italy (S.D.) (L.B.) (M.P.) (M.S.) (S.P.N.); 3 JC Self Research Institute, Greenwood Genetic Center, Greenwood, SC 29646, USA; 4 Clemson University School of Health Research, Clemson University, Clemson, SC 29634, USA; 5 Dermatology Unit “Daniele Innocenzi”, Department of Medical-Surgical Sciences and Biotechnologies, Sapienza University of Rome, Polo Pontino, 04110 Terracina, Italy (I.P.) (C.P.)

**Keywords:** gluten, gut, enteropathy, gluten-free diet, level of evidences

## Abstract

Celiac disease (CD) is an autoimmune enteropathy that primarily affects the small intestine and is characterized by atrophy of intestinal villi. The manifestations of the disease improve following a gluten-free diet (GFD). CD is associated with various extra-intestinal diseases. Several skin manifestations are described in CD patients. The present paper reviews all CD-associated skin diseases reported in the literature and tries to analyze the pathogenic mechanisms possibly involved in these associations. Different hypotheses have been proposed to explain the possible mechanisms involved in every association between CD and cutaneous manifestations. An abnormal small intestinal permeability seems to be implicated in various dermatological manifestations. However, most of the associations between CD and cutaneous diseases is based on case reports and case series and a few controlled studies. To better assess the real involvement of the cutaneous district in CD patients, large multicentric controlled clinical trials are required.

## 1. Introduction

Celiac disease (CD) is an autoimmune disorder that occurs in genetically predisposed subjects who develop an immune reaction to gluten [1]. CD primarily involves the small intestine. However, the clinical presentation can be characterized by both intestinal and extra-intestinal manifestations. The incidence of CD is up to 1% in the majority of populations. Genetic factors play an important role in the pathogenesis of CD. The almost totality of the patients with CD possess class II human leukocyte antigen (HLA) -DQ2 and -DQ8, or their variants. However, up to 40% of people with European and Asian origins carries these genes, indicating that the expression of these molecules is necessary, but not sufficient, to develop the disease [2].

The intestinal immune response to gluten is present in two sites: The lamina propria and the epithelium [3]. Both the lamina propria (adaptive) and intraepithelial (innate) immune responses are necessary for the generation of the pathological celiac lesion, but how these two processes interact is not clear [1]. The presentation of CD has shifted from the historically classic malabsorption pediatric symptoms in childhood to non-specific symptoms, which may be present also in adulthood. The symptoms classically include weight loss, chronic diarrhea, and failure to thrive. Non-specific symptoms are more common and include gastrointestinal manifestations, such as bloating, abdominal pain, constipation, as well as extra-intestinal manifestations, as osteoporosis, headache, iron deficiency, and chronic fatigue [4,5].

**Figure 1:**
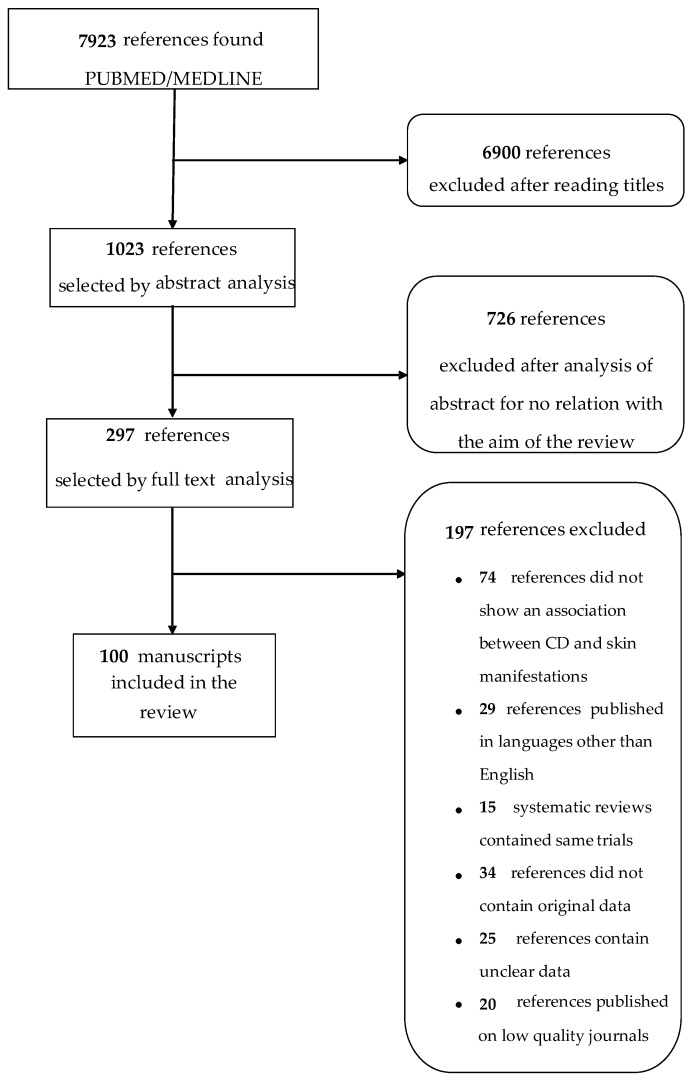
Flow chart of the identified and selected studies.

In the last years, skin diseases are acquiring more and more importance among the extra-intestinal manifestations of CD [4]. The aim of this review is to summarize the association between cutaneous diseases and CD. For this reason, searches were undertaken in the PubMed/Medline database in March 2019 using the following terms: A combined research of “celiac disease” and “skin”, “blistering diseases”, “cutaneous manifestations”, “pemphigus”, and other skin disorders. In this way, 7923 articles were found and 100 were selected as reported in Figure 1. In addition, the studies were rated using the Oxford Centre for Evidence Based Medicine 2011 and levels of evidence assigned to each association [6]. The assigned levels of evidence were discussed among members until consensus was reached (Table 1).

## 2. Blistering Diseases

Blistering diseases are characterized by the formation of bullae, blisters, and erosions on the cutaneous surface. Among them pemphigus, dermatitis herpetiformis and linear IgA bullous dermatosis may be related to CD.

### 2.1. Pemphigus

Pemphigus is a group of autoimmune diseases characterized by the formation of flaccid bullae and erosions of the mucosae and skin [7]. In 2014, an association among pemphigus, epilepsy, and CD was reported [8]. Various case reports associate pemphigus with the positive blood markers of CD, and in particular IgA anti-gliadin (AGA) and anti-endomysium (EMA) antibodies [8,9]. In this case, instauration of a gluten-free diet (GFD) may induce pemphigus disappearance [9].

### 2.2. Dermatitis Herpetiformis

Dermatitis herpetiformis (DH) or Duhring-Brocq disease is an inflammatory skin disease characterized by a chronic relapsing course and typical histopathological and immunopathological findings. It presents as symmetrical, grouped polymorphic lesions consisting of erythema, urticarial plaques, and papules involving the extensor surfaces of the elbows, knees, shoulders, buttocks, sacral region, neck, face, and scalp. Herpetiform vesicles may occur later and are often immediately excoriated, resulting in erosions, crusted papules, or areas of post-inflammatory dyschromia. Petechial or ecchymotic lesions may occur in the palmoplantar regions and are observed more frequently in children. DH is the most common extra-intestinal manifestation of CD in >85% of cases and it improves significantly with a GFD. In fact, DH and CD share the same HLA haplotypes, DQ2 and DQ8 [10]. Various authors showed the decreasing incidence rate of DH, along with a simultaneous rapid increase in CD. This fits the hypothesis that subclinical, undiagnosed CD is a prerequisite for the development of DH [11].

Moreover, patients with DH have elevated levels of IgA anti-transglutaminase antibodies both TG2 and TG3, which are the most sensitive and specific antibodies to be tested in patients with a suspected DH [12]. At present, a valid hypothesis is that the immune pathogenesis of DH starts from hidden CD in the gut with a TG2, and possibly also a TG3, autoantibody response and evolves into an immune complex deposition of high avidity IgA TG3 antibodies together with the TG3 enzyme in the papillary dermis. This seems to be due to the active TG3 enzyme in the aggregates resulting in covalent cross-linking of the complex to the dermal structures [13].

A small bowel biopsy shows typical CD alterations in almost all these patients, including: Partial-to-total villous atrophy, elongated crypts, decreased villus/crypt ratio, increased mitotic index in the crypts, increased intraepithelial lymphocyte (IEL) density, increased IEL mitotic index, infiltration of plasma cells, lymphocytes, mast cells, and eosinophils and basophils into the lamina propria [14]. The histology of DH is characterized by subepidermal vesicles and blisters associated with the accumulation of neutrophils at the top of dermal papillae. Sometimes, eosinophils can be found within the inflammatory infiltrate, making difficult the differential diagnosis with bullous pemphigoid. The histopathology of DH is not diagnostic as other bullous diseases, including linear IgA dermatosis and epidermolysis bullosa acquisita, which may show similar histologic findings [10]. It is important to obtain a skin fragment near the vesicle for histopathological analysis to identify neutrophilic micro-abscesses. Neutrophils and eosinophils on the top of dermal papillae may form the Piérard micro-abscess, which are typical of this dermatosis, but not pathognomonic. The direct immunofluorescence histology of perilesional skin affected is the gold standard to confirm the diagnosis. It shows the deposition of IgA1 in granular pattern in the lamina lucida of the basement membrane zone. The deposits of IgA in a linear pattern can be found in less than 5% of cases. Indirect immunofluorescence can be used to evaluate the presence of autoantibodies and circulating anti-gliadin, anti-endomysial, anti-reticulin IgA, and anti-epidermal transglutaminase antibodies (anti-tTG) [15]. The first-choice treatment of DH is a strict, life-long GFD which can lead to the resolution of cutaneous and gastrointestinal manifestations with relief in the itching and burning sensation of the vesicle-erythematous-papule. 

However, in the first month after the diagnosis or in the inflammatory phases of the disease, in which a GFD alone would not be enough to resolve the cutaneous manifestations, several drugs can be used, including dapsone, steroids or sulfones [10,15].

### 2.3. Linear IgA Bullous Dermatosis

Linear IgA bullous dermatosis (LABD) is a rare dermatosis characterized by small vesicles and erythematous papules. Pruritus is almost always present with an acute or gradual onset. Up to 24% of the patients affected by LABD also present gluten sensitivity enteropathy and they are responsive to a GFD [16]. LABD and CD often share the same HLA haplotype (i.e., A1, B8, DR3). Although very few case reports have been found in the literature, some authors suggested an association between LABD and gluten sensitivity [16].

## 3. Urticaria

Urticaria is characterized by intensely pruritic, raised wheals, with or without edema of the deeper cutis. It is usually self-limited, but can be chronic. Chronic urticaria (CU) is defined by recurrent episodes occurring at least twice a week for 6 weeks [17]. The association between CD and CU is widely debated. Ludvigsson and collaborators examined the association between CD and urticaria in 28,900 patients with biopsy-verified CD: 453 patients with CD and no previous diagnosis of urticaria developed urticaria and 79 of these 453 had CU. This confirms that CD is associated with urticaria, especially in its chronic form [17]. CU has been shown to have a genetic association with the human leukocyte antigen HLA-DQ8 alleles. Interestingly, HLAD-Q8 has an association with CD [18]. It has been shown that in CU, there are IgG autoantibodies that inappropriately activate mast cells. The inflammatory response generated in CD probably activates the cells that produce the IgG autoantibodies in CU. It has also been shown that, in patients affected by CD and urticaria, a GFD leads to complete remission of urticaria [19]. 

## 4. Hereditary Angioneurotic Edema

Hereditary angioneurotic edema (HANE) is an autosomal dominant genetic disease due to a C1-esterase inhibitor deficiency that leads to an overproduction of bradykinin, causing an increase in vascular permeability. It is characterized by recurrent, marked and diffuse swellings of the subcutaneous and submucosal tissues, painful abdominal attacks, and laryngeal edema with airway obstruction. The symptoms in HANE might be mediated by the activation of the complement system and recent clinical data indicate that the major mediator of angioedema is bradykinin [20]. The therapy consists mainly in the C1-esterase inhibitor concentrate and fresh frozen plasma [20]. The association between CD and hereditary angioneurotic edema, was first reported by Farkas and co-workers [21]. The activation of the pathways of the complement system plays an important role in the pathogenesis of CD and HANE. Gluten ingestion can stimulate complement activity as well as HANE is characterized by an activation of the classic complement pathway [22]. Screening hereditary angioedema patients for CD is warranted if abdominal attacks or neurological symptoms persist despite adequate management. Complement testing is recommended whenever abdominal symptoms persist despite the histological and serological remission of gluten-sensitive enteropathy after the introduction of a GFD [22]. 

## 5. Atopic Dermatitis

Atopic dermatitis (AD) is a chronic inflammatory skin disease associated with a heterogeneous group of symptoms and signs. Cutaneous signs and symptoms include erythema, lichenification, prurigo nodules and itch. AD affects 40 million individuals worldwide, and its prevalence is still increasing. Notably, AD appears to be more prevalent among children under five years of age with a decrease in adulthood. The onset of AD occurs primarily in childhood and is thought to precede allergic disorders mediated by IgE sensitization to environmental antigens in the patient affected by atopic triad, as well as AD, asthma, and allergic rhino-conjunctivitis. The complex interaction between genetics, environmental factors, microbiota changes, skin barrier deficiency, immunological derangement, and possibly autoimmunity, contributes to the development of this skin disease [23]. AD has also been linked to CD. Ress and coworkers analyzed the prevalence of CD in children with AD compared with a general pediatric population and showed a four-fold greater risk of developing CD in patients with AD (OR, 4.18; 95% CI, 1.12–15.64). This association may be explained, considering the common cytokine pathways between the two diseases and the screening for CD in patients with AD must be considered in order to prevent the long-term complications of CD [24]. 

## 6. Cutaneous Vasculitis

Vasculitis is an inflammatory process affecting the vessel wall and leading to its compromise or destruction with subsequent ischemic and hemorrhagic events. Cutaneous vasculitis is generally characterized by petechiae, palpable purpura and infiltrated erythema indicating dermal superficial, small-vessel vasculitis, or less commonly by nodular erythema, deep ulcers, livedo racemosa, and digital gangrene implicating deep dermal or subcutaneous, muscular-vessel vasculitis [25]. A skin biopsy, extending to subcutis and taken from the earliest, most symptomatic, reddish or purpuric lesion, represents the gold standard for the diagnosis of cutaneous vasculitis. The association between CD and cutaneous vasculitis has been described in several reports [19]. Leukocytoclastic vasculitis (LV), also known as hypersensitivity vasculitis, is a small vessel vasculitis, and it is thought to result from the deposition of circulating immune complexes into the vessel walls, specifically in dermal post-capillary venules, activating the complement pathway. LV is usually limited to the skin and may manifest as palpable purpura, maculopapular rash, bullae, papules, nodules, or ulcers [26]. Meyers et al. reported a case of a young woman, affected by uncontrolled CD, who presented cutaneous LV with the remission of skin lesions after the treatment with a GFD. The coexistence of these two entities might be related to increased intestinal permeability. Exogenous antigens may permeate the damaged CD mucous in larger quantities than normal. This may explain the elevated gluten fraction antibody titer. Moreover, circulating immune complexes are well documented in CD. They probably originate because of the impaired phagocytic function of the reticular endothelium system and subsequently they are deposited in the skin [27,28]. The treatment with a GFD may improve CV lesions in cases associated with CD [28].

## 7. Erythema Nodosum

Erythema nodosum (EN) is the most common form of panniculitis. It classically presents as tender, warm, erythematous subcutaneous nodules on the bilateral pretibial areas. Although it can occur in both sexes and at any age, it affects predominantly young women [29]. EN associated with CD was reported three times since 1991. It was proposed that the augmented intestinal permeability to various antigens may provoke the skin hypersensitivity reaction [30]. Generally, EN resolves within 8 weeks but as an active disease, it may last up to 18 weeks, and even for a longer period when the antigenic stimulus persists. Furthermore, it has been reported in the literature that CD may coexist with sarcoidosis which is a common cause of EN [31]. EN associated to CD may be far more common than expected. All patients with recurrent or persistent EN of unknown origin should be screened for an underlying CD.

## 8. Erythema Elevatum Diutinum

Erythema elevatum diutinum (EED) is a rare, chronic and treatable skin condition. The etiology of the disease is unknown, but it has been suggested to be related to high circulating levels of antibodies formed in response to repeated infection. It has many histological mimics and is often associated with several systemic diseases [32]. EED is considered to be a variant of leukocytoclastic vasculitis clinically manifesting as asymptomatic to painful erythematous papules, plaques or nodules, which are usually distributed symmetrically on the extensor surfaces of extremities. It is rarely accompanied by systemic features other than arthritis [32]. Rodriguez-Serna et al. described for the first time, the association between CD and EED in an 11-year-old girl, considering it not a coincidence. With the beginning of the GFD, the cutaneous lesions disappeared and anti-gliadin antibody levels returned to normal. Both conditions have an immune basis in which the increase in IgA appears to play an important role [33]. The pathogenesis of EED is characterized by immune complex deposition, resulting in complement activation, neutrophilic infiltration, and the release of destructive enzymes [32]. Tasanen and others described the presence of granular deposits of IgA and C3 at the derma-epidermal junctions in the affected skin of a patient who presented with EED and CD [34]. Therefore, it is important to evaluate the presence of CD in patients with EED. 

## 9. Necrolytic Migratory Erythema

Necrolytic migratory erythema (NME) is the acronym used for the first time by Wilkinson, to describe the characteristic cutaneous pathology related to glucagonoma. It presents with red-blotch rashes, irregular edges and with vesicles that can be intact or followed by crusting erosions. Frequently, it affects the skin surrounding the lips and upper limbs, more rarely the lower limbs, the abdomen, the groin, the perineum and the buttocks. The affected areas may appear dry or fissured. Initially, the lesion may be exacerbated by pressure or trauma. The phases of lesion development are observed synchronously. Most of the patients have angular cheilitis, stomatitis, glossitis, alopecia, and gastrointestinal symptoms, such as weight loss and diarrhea [35]. NME develops in approximately 70% of patients with glucagonoma syndrome. The etiology is still unclear although a very strong relationship has been noted between hypoaminoacidemia, zinc and a fatty acids deficit due to malabsorption with the severity of NME. A further relationship with NME has been shown in inflammatory autoimmune diseases, such as CD. In fact, the patients affected by CD, who developed the NME, substantially improved the severity of the latter by following a GFD [36].

## 10. Psoriasis

Psoriasis is a very common chronic inflammatory disease characterized by scaly, well-demarcated, erythematous plaques affecting up to 2% of the general population. The lesions are distributed on different parts of the body, in particular the elbows, knees, trunk, hands and feet [37]. The disease-related quality of life is significantly reduced in patients affected by psoriasis [38]. Psoriatic patients are more likely to have other concomitant autoimmune diseases, such as ulcerative colitis and Crohn disease. CD may be considered as part of these groups. A recent study showed that psoriatic patients have a 2.2-fold risk of being diagnosed with CD compared to healthy controls [39]. A metanalysis showed that IgA AGA, were positive in approximately 14% of psoriatic patients versus 5% of matched controls. Moreover, there was a correlation between the CD antibody positivity and the severity of psoriatic manifestations. Interestingly, the elevated CD antibodies did not always lead to a biopsy-confirmed diagnosis of CD, suggesting that an association between psoriasis and gluten sensitivity, marked by antibody positivity, may be present even in the absence of CD [40]. Psoriasis and CD share different biological mechanisms. Various susceptibility loci are common to both diseases, in particular genes regulating innate and adaptive immune response, such as *RUNX3*, *TNFAIP3*, *SOCS1*, *ELMO1*, *ETS1*, *ZMIZ1*, *UBE2L3.34-36*, and *SH2B3* [41]. Psoriasis and CD have also both been linked to dysregulation in the pathways of Th1, Th17 cells, gamma-delta t-cells and an increased intestinal permeability [42]. Although there are no big clinical trials regarding this argument, a GFD seems to be beneficial for psoriatic patients. Two small clinical trials showed a decrease in serological markers of CD after a GFD and one showed a significant reduction in the psoriasis area severity index. Three case reports also documented the resolution of psoriasis after a GFD [40]. There is also an Italian multicenter study showing that 7 out of 8 patients affected by CD and psoriasis who underwent a GFD showed a significant improvement in the psoriasis area severity index, suggesting a role of gluten in the pathogenesis of both diseases [43].

## 11. Vitiligo Disease

Vitiligo is an acquired disease characterized by skin depigmentation with the formation of circumscribed white macules, without melanocytes. It predominantly affects the photoexposed areas of the body and the darker phototypes. The disease affects approximately 1% of people in the world and it is often a pathology with inherited characteristics [44]. The etiology is complex and not entirely known. The present dogma suggests that several genetic factors render the melanocyte fragile and susceptible to apoptosis, thus predisposing individuals to developing vitiligo [45]. The association between vitiligo and autoimmune diseases has not yet been fully explained, but genetic data have provided important insights. The susceptibility genes that were identified encode components of the immune system, supporting the hypothesis of a deregulated immune response in vitiligo (HLA class I and II, PTPN22, IL2Rα, GZMB, FOXP3, BACH2, CD80, and CCR6) [46]. The relationship between vitiligo and CD is controversial. The study of Shahmoradi et al. analyzed the frequency of celiac autoantibodies in a group of vitiligo patients compared with the control, involving 128 individuals, 64 vitiligo patients and 64 individuals as the control group [47]. Both IgA EMA and anti-transglutaminase antibodies were measured by the ELISA method in the serum of all participants. The serum of the two vitiligo patients was positive for antibodies. All control groups were seronegative for these antibodies (*p* < 0.05). This study may indicate that both of these autoimmune diseases may be stimulated by a common signal in the immune system that is triggered by a gluten rich diet [47].

## 12. Behçet’s Disease

Behçet’s disease (BD) is a chronic-relapsing inflammatory pathology of unknown etiology, characterized by frequent episodes of oral and/or genital ulcers, iritis, associated with various systemic manifestations such as joint, cutaneous and vascular lesions [48]. Behcet’s disease can be traced back to an abnormal immune response due to the exposure to a particular antigen in individuals with a genetic predisposition. The onset is between 10 and 30 years with a clear prevalence of the male sex 5–10 times more than women [49]. The stories of oral ulcers and enamel defects have been reported in approximately 25% of patients with CD [50]. There are a few studies in the literature that have elucidated a possible association between BD and CD. Caldas et al. evaluated the association between the two diseases in a 40-year-old woman who presented with asymmetric polyarthralgia, loss of weight, anemia, oral recurrent aphthas (>3/year) and genital ulcerations, inflammatory lower back pain, bowel bleeding and abdominal colic. The biopsy confirmed the diagnosis of CD and a GFD was applied with clinical improvement [51]. Ultimately, it would be desirable for patients with BD to be better investigated for a possible undiagnosed CD.

## 13. Aphthous Stomatitis

Recurrent aphthous stomatitis (RAS) is a common clinical condition that produces painful ulcerations in the oral cavity. RAS is characterized by multiple recurrent small, round, or ovoid ulcers with circumscribed margins, erythematous haloes typically first presenting in childhood or adolescence. RAS has been recognized for many years as a symptom of CD. A recent meta-analysis showed that celiac patients have greater frequency of RAS (OR = 3.79; 95% CI = 2.67–5.39). RAS patients should be considered at-risk subjects, even in the absence of any gastrointestinal symptoms and should therefore undergo a diagnostic procedure for CD [52]. The etiopathology of RAS is unclear. It is not known whether RAS lesions are directly influenced by the gluten sensitivity disorder, or if they are related to hematinic deficiency with low levels of serum iron, folic acid, and vitamin B12 or trace element deficiencies due to the malabsorption in patients with untreated CD.

## 14. Oral Lichen Planus

Oral cavity may be involved in CD. The oral manifestations associated to CD are recurrent aphthous stomatitis, glossitis, dental enamel defects, angular cheilitis, burning mouth and oral lichen planus. Oral lichen planus (OLP) is a chronic inflammatory disorder that affects the oral mucosa, gums and tongue with a spectrum of clinical manifestations, including atrophic, erosive, keratotic and ulcerative lesions [53]. OLP, as CD, is characterized by a T-cell autoimmune pathogenesis. Cigic et al. evaluated the prevalence of CD in patients with OLP compared to the controls. In this study, AGA and anti-tTG, were evaluated in 56 OLP patients [54]. CD was diagnosed in eight OLP patients (14.29%) and six OLP patients (10.71%) were positive for IgA Ttg. This confirms the increased frequency of CD in OLP patients [54]. Some erosive, atrophic and ulcerative oral lesions may be caused by underlying haematinic deficiencies associated to the nutrient malabsorption status of CD patients [55].

## 15. Dermatomyositis

Dermatomyositis (DM) is a rare autoimmune disease that preferentially affects the skin, lungs, muscles and blood vessels and is characterized by proximal and symmetrical muscle weakness with inflammation and damage to the parenchymal cells, causing erythematous and edematous skin manifestations. Usually this is an idiopathic disease. However, it can be associated with other concomitant connective tissue pathologies [56]. It can sometimes occur as a paraneoplastic syndrome associated with gastrointestinal or ovarian malignancies [57]. DM is characterized by the presence of autoantibodies, even if the mechanisms of inflammation and cell damage still remain unclear today [58]. The inflammatory infiltrate in the muscular tissue of DM is represented by T CD4+, B cells and dendritic cells which are preferentially localized around perimysial blood vessels and peripheral areas [59,60]. The inflammation causes damage to the parenchyma and to the blood vessels, so the histological examination shows the presence of a perimysial atrophy, a predominantly perivascular and interfascicular inflammation [59]. The typical histological findings on skin biopsy are vacuolar interface dermatitis with apoptosis, necrotic keratinocytes, and perivascular lymphocytic infiltrate and mucin deposition in the dermis. The inflammatory infiltrate in DM muscle tissue consists of CD4+ T cells, B cells, and dendritic cells that primarily concentrate around perifascicular areas and perimysial blood vessel [58]. Several case reports have highlighted the association between DM and CD [57,61,62,63,64]. 

## 16. Porphyria

Porphyria, derived from the ancient Greek word “porphura”, that is purple, is a group of nine rare diseases: Acute intermittent porphyria (AIP), hereditary coproporphyria (HCP), variegated porphyria (VP), delta-aminolevulinic acid dehydratase deficiency porphyria (ADP), porphyria cutanea tarda (PCT), hepatoerythropoietic porphyria (HEP), congenital erythropoietic porphyria (CEP), erythropoietic protoporphyria (EPP), and X-linked protoporphyria (XLP), characterized by metabolic alterations and caused by malfunctioning of the enzymes involved in the biosynthesis of heme with the accumulation and excretion of porphyrins and their precursors in tissues [65]. The enzyme activity decreases and involves an overproduction of heme precursors, except in XLP [66]. HEP rarely cause clinical manifestations before puberty, as opposed to EPP that manifests symptomatically in the early stages. The precursors of heme of various types accumulate in the liver or bone marrow, which are the most active tissues for the production of heme. All these mechanisms underlie the classification of porphyria as hepatic or erythropoietic. It generally occurs with cutaneous manifestations due to phototoxicity, or with neurological changes such as acute attacks. Based on these differences, the porphyrias are classified as acute or cutaneous. Phototoxicity can occur in all porphyrias, except in ADP and AIP [65]. The urine of patients with this condition may be dark or reddish due to the presence of an excess of porphyrins and related substances. These substances are photosensitizing, and their accumulation causes skin fragility, bullae, scars, hirsutism and the characteristic pigmentation on the photo-exposed areas. This is due both to the release of mediators by leukocytes and mast cells and to the activation of the complement, determining the inflammatory response after photoexposure [67]. Some studies, such as Twaddle and collaborators, have shown a random diagnosis of CD in patients with porphyria [68]. In fact, CD is often associated with DH, underlining some common characteristics of the latter with porphyria. In the VP, the accumulation of 5-aminolevulinic acid and of porfobilinogen provokes both gastrointestinal manifestations and an acute neuropsychiatric syndrome. In this regard, it is important to underline how other works have shown, in patients suffering from CD, a reduction of acute attacks of VP during the GFD [69].

## 17. Alopecia Areata

Alopecia areata (AA) is a complex, polygenic pathology with autoimmune etiology, which results in the transitory, non-scarring hair loss and the preservation of the hair follicle. By prevalence, it represents the second cause of non-scarring alopecia [70,71]. Clinically, hair loss in alopecia areata manifests itself through very different models. The most frequent pattern is characterized by a small annular or irregular lesion (alopecia areata to patches), usually localized on the scalp, being able to progress until total hair loss, and in this case, the discussion is about total alopecia, associated or not with total loss of all body hair [70]. A biopsy performed on the affected skin shows a lymphocyte infiltrates around the bulb or in the lower part of the hair follicle, thus it suggests an immunological etiology like CD. Recent studies focused on chromosome 6 and more specifically, on the HLA region as the most probable region for the genes that regulate susceptibility to AA [72]. Linkage studies based on a genome-wide association study analysis, have identified an association with many chromosomes and show that AA is a very complex polygenic pathology [70,73]. Both in the AA and in the CD, the presence of organ-specific autoantibodies has been demonstrated [70], with infiltration of T lymphocytes on the lesion site [74]. AA can occur at any age, although most patients develop the disease before the age of 40, with an average age of onset between 25 and 36 years. Early onset between 5 and 10 years, presents itself as a more severe subtype, as an alopecia universalis [75]. Hallaji et al. found that the prevalence of anti-gliadin antibodies in patients with AA had a proportion of 18:100 [76]. In several studies [71,76,77], it has been hypothesized the existence of a CD not diagnosed in patients with AA which improves during a GFD. These positive effects of a GFD on AA, have been associated with the normalization of the immune response [76]. The chronic recurrent phases of CD can be observed during the normal clinical course of AA, and it was noted that patients who followed a GFD regimen showed complete regrowth of hair and other body hair, without highlighting a further recurrence of AA during the follow-up [78]. However, in the study by Mokhtari et al., the frequency distribution of all celiac autoantibodies has been analyzed in patients with AA. The results led to the conclusion that the various biological tests used for the research of subclinical CD do not provide sufficient clear evidence to make the diagnosis of gluten intolerance in patients with AA and other diagnostic approaches are needed [79].

## 18. Rosacea

Rosacea is an inflammatory skin condition, more frequent in women and primarily characterized by persistent or recurrent episodes of centrofacial erythema [80]. The pathophysiology is not completely understood, but the dysregulation of the immune system as well as changes in the nervous and vascular systems have been identified. Rosacea shares genetic risk loci with autoimmune diseases, such as type 1 diabetes mellitus and CD [81]. One study showed that women with rosacea had a significantly increased risk of CD. In a nationwide cohort study, the prevalence of CD was higher among patients with rosacea when compared to the control subjects (HR = 1.46, 95% CI = 1.11–1.93). However, the pathogenic link is not known. Gastrointestinal symptoms in patients affected by this dermatological condition should warrant clinical suspicion of CD [82].

## 19. Acquired Hypertrichosis Lanuginosa

Acquired hypertrichosis lanuginose (AHL) is a rare cutaneous manifestation that often underlies the presence of neoplastic pathologies, particularly in the elderly. It is considered as a paraneoplastic manifestation of organic tumor forms, such as a tumor of the gastrointestinal tract, of the lung, of the uterus, of the breast, and often it is indicator of a poor prognosis. It can also be associated with lymphomas. [83]. The etiology is not clear. Corazza and coworkers observed a case of hypertrichosis in a patient suffering from CD [84]. The patient developed a tumor shortly after the diagnosis of CD, confirming that the ACL could represent the unknown tumor spy [84]. However, due to the insufficient data present in the literature, it is not possible to establish a certain correlation.

## 20. Pyoderma Gangrenosum

Pyoderma gangrenosum (PG) is a rare ulcero-necrotising and neutrophilic dermatosis, whose pathogenesis is unknown. Generally, the main manifestation is a sterile pustule, that rapidly develops into a painful ulcer from the erythematous border [85]. The diagnosis is often a challenge for the clinician and it is often achieved by exclusion. In the literature, many studies have shown the association between inflammatory bowel diseases and in particular, Crohn’s disease and ulcerative colitis, and skin manifestations including PG [86]. Weizman et al. have described one of the largest case series of PG among patients with inflammatory bowel disease (IBD) [87]. Moreover, it was observed that the female sex and young age at diagnosis of IBD are predictive factors involved in the development of main cutaneous manifestations [88]. Furthermore, refractory CD resistant to steroids and immunosuppressive drugs has been reported to be associated to PG [89]. The appearance of a pustule transforming to an ulcer in a patient with CD should always arise the suspect for PG [87].

## 21. Ichthyosiform Dermatoses

Ichthyosiform dermatoses are a group of a skin disorders characterized by clinically evident thickening of the stratum corneum, dry skin and often erythroderma. The term ichthyosis derives from the Greek “ichthys” that means fish, referring to the cutaneous scaling characteristic of these disorders, which is said to resemble the scales of a fish. The attention for this pathology is quite recent. In fact, the first international ichthyosis conference classification was approved in 2009 [90]. The first case report associating the ichthyosis with CD was observed by Menni and co-workers. They reported the clinical history of a twenty-nine year-old woman, who presented ichthyosiform skin manifestations [91]. After just over 10 years, another case that showed this association was reported. In this case, the patient was younger than the first case, but also affected by osteoporosis and secondary hyperparathyroidism. Although the GFD did not allow a complete disappearance of the cutaneous manifestations, it favored an improvement in the quality of life [92].

## 22. Pellagra

Pellagra is a rare disease caused by niacin deficiency and characterized by a classical triad: Dermatitis, dementia and diarrhea, known as “3D” [93]. The skin signs are the first to appear in over 80% of cases. Initially, itchy and erythematous lesions appear on the area of photo-exposed skin and generally, they are bilateral and symmetrical lesions localized on the dorsum of the hands, arms, face and neck. Subsequently, the gastrointestinal and neurological symptoms occur [93]. In 1937, a case was published of a 15-month-old child with skin manifestations compatible with a non-classical form of pellagra and the simultaneous presence of gastrointestinal disorders related to CD [94]. More than 60 years later, Schattner described another case of a 70-years-old man with a pellagra-like syndrome due to CD [95]. No other cases have been reported, determining a weak association between the two conditions. 

## 23. Generalized Acquired Cutis Laxa

Cutis laxa (CL), also called elastolysis or dermatomegaly, includes a heterogenous group of disorders that affects the elastic tissue and it is characterized by the presence of loose and redundant skin, caused by a reduced number and abnormal properties of elastic fibers in the derma [96]. The etiopathogenesis is still not fully known; it can be congenital or acquired [97]. Only one case in the literature shows the coexistence of acquired CL and CD. In this case, the clinical suspicion of CL was confirmed histologically and analyzed through direct immunofluorescence. The latter has highlighted the presence of IgA deposits in the papillary dermis. It is possible to hypothesize that the IgA deposit constitutes the base of the pathophysiological link between the two diseases [71,98].

## 24. Skin Malignancies

The variations of the incidence of the skin malignancies in patients affected by CD is a very interesting topic. A study by Ilus et al. studied a population of 32,439 adult celiac patients to evaluate the relative risk for each kind of malignancies [99]. Among cutaneous cancer, a major incidence was registered for basal cell carcinoma and a slightly minor incidence for melanoma [99]. There is instead a known association between CD and lymphomas [99], and cutaneous lymphomas seem also associated to CD. Various case reports show this association, the last one being reported in 2016. The association of cutaneous lymphoma with CD may be determined by a lymphocytic stimulation carried out by the presence of a constant antigen, such as gluten, in the gastrointestinal tract. Some researchers [100] suggested that in predisposed individuals, the resulting dysplastic T cells may migrate into cutis, causing cutaneous T cell lymphoma. The adherence to a GFD decreases the risk for malignancy. 

## 25. Conclusions

CD is an autoimmune enteropathy associated with several extra-intestinal diseases, including various skin manifestations. Different hypotheses have been proposed to explain the possible mechanisms involved in every association between CD and cutaneous manifestations. An abnormal small intestinal permeability seems to be implicated in various dermatological manifestations. The inability of the small intestine to operate as a barrier may allow a major penetration of exogenous antigens with a consequent immunological response that leads to vascular alterations and to malabsorption with secondary vitamin and amino acidic deficiency. However, on the basis of the revision of the literature by the levels of evidence, it can be concluded that only a few associations are very strong and in particular, DH and psoriasis. The other associations between CD and skin diseases are based on case-reports and case series, with very few multicentric controlled studies. In this way, to better assess the involvement of the cutaneous district in CD, large multicentric controlled studies are required. Nevertheless, screening for CD in patients suffering from chronic cutaneous diseases seems to be justified considering that a GFD can significantly improve both gastrointestinal and systemic symptoms.

## Figures and Tables

**Table 1:** Level of evidences of the association between celiac disease and skin disorders.

Disorders Associated	Level of Evidence
Pemphigus	4
Dermatitis herpetiformis	1A
Linear IgA bullous dermatosis	4
Urticaria	2B
Hereditary angioneurotic edema	4
Atopic dermatitis	4
Cutaneous vasculitis	4
Erythema nodosum	4
Erythema elevatum diutinum	4
Necrolytic migratory erythema	4
Psoriasis	1A
Vitiligo disease	3B
Stomatous Aphtitis	2A
Behçet’s disease	4
Oral lichen planus	3B
Dermatomyositis	4
Porphyria	4
Rosacea	2B
Alopecia areata	3B
Acquired hypertrichosis lanuginosa	5
Pyoderma gangrenosum	4
Ichthyosiform dermatoses	4
Pellagra	5
Generalized acquired cutis laxa	5
Skin malignancies	2B

Levels of evidence: 1A Systematic Reviews of Randomized Control Trials; 1B Individual RCT; 2A Systematic Reviews of cohort studies; 2B Individual cohort study; 3A Systematic Reviews of case-control studies; 3B Individual Case-Control Study; 4 Case-series (and poor quality cohort and case-control studies); 5 Expert opinion without explicit critical appraisal.
